# Treatment Outcomes of Clazosentan Use During the Perioperative Period for Subarachnoid Hemorrhage

**DOI:** 10.7759/cureus.79497

**Published:** 2025-02-23

**Authors:** Eitaro Okumura, Kohei Nakaya, Kunitoshi Otsuka, Hiroyuki Jimbo

**Affiliations:** 1 Department of Neurosurgery, Tokyo Medical University Hachioji Medical Center, Hachioji, JPN

**Keywords:** aneurysmal subarachnoid hemorrhage, cerebral vasospasm, clazosentan, delayed cerebral ischemia (dci), symptomatic pulmonary edema

## Abstract

Background

Fasudil hydrochloride hydrate has been traditionally administered in the perioperative management of aneurysmal subarachnoid hemorrhage (aSAH) in Japan for the prevention of delayed cerebral ischemia (DCI) secondary to cerebral vasospasm. While clazosentan, a selective endothelin receptor antagonist, was introduced in April 2022 as an alternative therapeutic option for the same indication, comparative data regarding the therapeutic effectiveness between these agents remains limited. Therefore, this study investigated the differences in treatment outcomes between traditional fasudil hydrochloride hydrate and clazosentan in the perioperative management of aSAH.

Materials and methods

We retrospectively analyzed aSAH cases treated at our hospital from April 2020 to April 2024. Cases were stratified into either the conventional (fasudil hydrochloride hydrate) or clazosentan group. The primary endpoint was the frequency of DCI associated with cerebral vasospasm. The secondary endpoints were moderate or severe cerebral vasospasm within 14 days of aSAH onset, frequency of rescue therapy, modified Rankin scale (mRS) ≤3 at discharge and hospital stay duration. The postoperative incidence of symptomatic pulmonary edema and mortality assessed safety.

Results

The study analyzed 104 cases, 61 in the conventional group and 43 in the clazosentan group. The frequency of DCI did not differ between the conventional and clazosentan groups (three cases vs. one case, respectively). Similarly, no significant differences were observed in moderate or severe cerebral vasospasm, rescue therapy, or hospital stay duration. The conventional group had 29 cases with mRS ≤3 at discharge compared with 31 in the clazosentan group. A significantly higher incidence of symptomatic pulmonary edema was observed in the clazosentan group, with 15 cases vs. eight cases in the conventional group. No difference was observed in mortality at discharge.

Conclusions

We compared treatment outcomes between fasudil hydrochloride hydrate and clazosentan for aSAH. While clazosentan showed a non-significant trend toward lower DCI frequency, it was associated with increased symptomatic pulmonary edema. Given the study's limitations, larger-scale research with matched baseline characteristics is needed to definitively evaluate these agents' comparative efficacy.

## Introduction

Delayed cerebral ischemia (DCI) due to cerebral vasospasm occurs in 20-40% of aneurysmal subarachnoid hemorrhage (aSAH) cases, leading to poor prognosis [1,2]. Traditionally, the rho kinase inhibitor fasudil hydrochloride hydrate has been used for vasospasm prevention in Japan. Fasudil hydrochloride hydrate is a potent vasodilator that was discovered and developed in Japan [3]. While this is the conventional therapy, clazosentan is a selective endothelin receptor subtype A (ETA) antagonist. Endothelin-1 is a potent, long-lasting endogenous vasoconstrictor implicated in vasospasm's pathogenesis [4,5]. In aSAH, the group treated with clazosentan showed a significantly lower incidence of morbidity/mortality events related to cerebral vasospasm compared with the placebo group [6]. Consequently, clazosentan was approved in Japan in April 2022 to prevent and treat cerebral vasospasm after aSAH. The Japan Stroke Society’s 2023 revised guidelines confer clazosentan with a grade B recommendation [7]. However, pulmonary edema is a specific complication associated with clazosentan use [8], requiring a different management strategy from conventional fluid administration. This study described fluid management practices with clazosentan at our hospital and compared the treatment outcomes between the perioperative use of clazosentan and fasudil hydrochloride hydrate in aSAH.

## Materials and methods

Study design

This study was a single-center, retrospective observational study conducted from April 2020 to April 2024. The criteria for inclusion were (1) patients aged ≥20 years; (2) patients with aSAH (including World Federation of Neurosurgical Societies (WFNS) classification grades I-V); (3) patients who underwent surgery within 72 hours of aSAH onset; and (4) continued administration of fasudil hydrochloride hydrate or clazosentan for 14 days from the day after surgery. The exclusion criteria were (1) cases with pre-existing cerebral vasospasm, as identified through preoperative cerebral angiography; (2) cases without cerebral vascular evaluation within 14 days; and (3) cases with extensive cerebral infarction due to surgical complications.

Ethical considerations

This study was conducted in accordance with the principles of the Declaration of Helsinki and approved by the Institutional Review Board of Tokyo Medical University (approval number: T2024-0011). Reporting followed the STROBE (Strengthening the Reporting of Observational Studies in Epidemiology) criteria. The requirement for informed consent was waived because the analysis used anonymous clinical data obtained after each patient agreed to undergo the assigned treatment and provided written consent. Consent for this study was obtained via the opt-out method publicized through a poster, which the Institutional Review Board approved.

Postoperative management protocol for vasospasm

Prior to clazosentan approval in Japan, vasospasm management was primarily based on fasudil hydrochloride hydrate and ozagrel sodium administration, as recommended by the Japan Stroke Guidelines 2021. We modified the perioperative vasospasm management protocols for the conventional (fasudil hydrochloride hydrate) and clazosentan groups (Table 1).

**Table 1:** Perioperative vasospasm management protocols CTA: computed tomography angiography, MRA: magnetic resonance angiography, PTA: percutaneous transluminal angioplasty

	Clazosentan protocol	Conventional protocol
Systolic blood pressure	Normotension (100–140 mmHg)	Normotension (100–140 mmHg)
Water balance	Normovolemia with a slightly negative balance target of −500 to +500 mL total balance from admission	Normovolemia with a slightly positive balance target of 0–1000 mL total balance from admission
Intravenous administration	continuous intravenous clazosentan (10 mg/h for 14 days)	intravenous fasudil hydrochloride hydrate (30 mg three times a day for 14 days)
Oral medicine	Cilostazol and/or statins	Cilostazol and/or statins
Image assessment	CTA, MRA, or cerebral angiography was performed in all cases on the 7th and 14th postoperative days	CTA, MRA, or cerebral angiography was performed in all cases on the 7th and 14th postoperative days
Rescue therapy	Intra-arterial fasudil hydrochloride hydrate or PTA for the targeted vessels	Intra-arterial fasudil hydrochloride hydrate or PTA for the targeted vessels
Drainage	Ventricular and/or spinal drainage	Ventricular and/or spinal drainage

The common points between the two groups were as follows: systolic blood pressure management aimed at normotension (100-140 mmHg); administration of cilostazol and statins at the attending physician’s discretion; and drainage methods (ventricular or spinal drainage) also at the physician’s discretion. Rescue therapy involved intra-arterial fasudil hydrochloride hydrate or percutaneous transluminal angioplasty for the targeted vessels. The differences between the groups were as follows: The conventional group administered intravenous fasudil hydrochloride hydrate (30 mg three times a day for 14 days) and managed normovolemia with a slightly positive balance target of 0-1000 mL total balance from admission. The clazosentan group administered continuous intravenous clazosentan (10 mg/h for 14 days) and managed normovolemia with a slightly negative balance target of −500 to +500 mL total balance from admission.

Image assessment

Computed tomography angiography, magnetic resonance angiography, or cerebral angiography were performed in all cases on the 7th and 14th postoperative days. Moderate or severe cerebral vasospasm was defined as ≥34% vessel narrowing compared with the initial vascular evaluation. DCI was defined as the presence of new cerebral infarction in territories corresponding to moderate or severe angiographic vasospasm without consideration of clinical deterioration. Blinded reviewers evaluated imaging assessments.

Endpoints

The primary endpoint was the frequency of DCI related to cerebral vasospasm. Secondary endpoints included the frequency of moderate or severe cerebral vasospasm within 14 days of aSAH onset, rescue therapy frequency, cases with modified Rankin scale (mRS) ≤3 at discharge, and the duration of the hospital stay. Safety endpoints included the frequency of symptomatic pulmonary edema and mortality at discharge.

Statistical analysis

Data are expressed as median (interquartile range) for continuous variables and number (%) for categorical variables. The Mann-Whitney U test was used to analyze continuous variables, and Fisher’s exact test and Pearson’s chi-square test were used for categorical variables. We analyzed the relationship between the patients’ baseline characteristics and their treatment outcomes. The significance level was set at p<0.05. All statistical analyses were performed using SPSS Statistics version 29 (IBM Corp. Released 2023. IBM SPSS Statistics for Windows, Version 29.0.2.0 Armonk, NY: IBM Corp).

## Results

Patient characteristics

**Figure 1:**
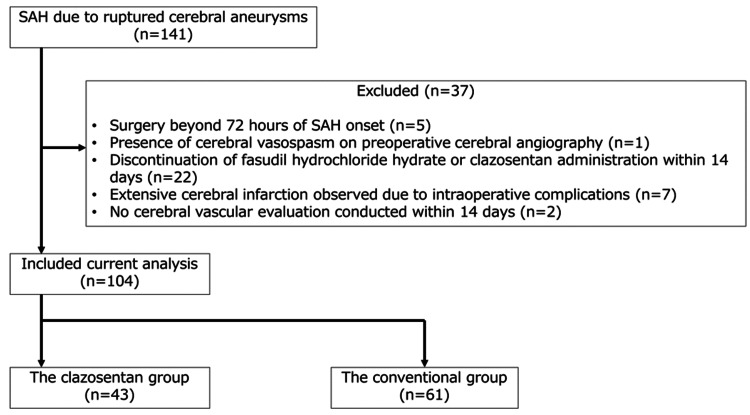
Patient extraction results A flowchart shows the selection of patients with aSAH for inclusion in this study. We identified 141 cases of aSAH due to a ruptured cerebral aneurysm, with 86 cases treated with fasudil hydrochloride hydrate and 55 cases with clazosentan. After excluding cases that met the exclusion criteria, the study included 61 cases in the conventional group and 43 in the clazosentan group. aSAH: aneurysmal subarachnoid hemorrhage

Out of 141 cases of aSAH treated during the study period, 104 were included. Of these, 61 were in the conventional group, and 43 were in the clazosentan group (Figure 1). The patient characteristics are presented in Table 2. A comparison between the conventional and clazosentan groups showed no significant differences in age, medical history, aneurysm location, surgical approach, intracranial hemorrhage, intraventricular hemorrhage, the use of statins and cilostazol, or drainage methods. However, the clazosentan group included a significantly higher proportion of women and had a higher incidence of mild cases (WFNS grades I and II). In contrast, the conventional group had a significantly higher number of severe cases (WFNS grades IV and V) and a higher incidence of Fisher group four cases.

**Table 2:** Patient characteristics Data were expressed as median (interquartile range) for continuous variables and frequencies (%) for categorical variables. The Mann–Whitney U test was used to analyze continuous variables, and Fisher’s exact test and Pearson’s chi-square test were used for categorical variables. WFNS: World Federation of Neurosurgical Societies classification grade, MCA: middle cerebral artery, ACA: anterior cerebral artery, ICA: internal cerebral artery, BA: basilar artery, VA: vertebral artery, ICH: intracranial hemorrhage, IVH: intraventricular hemorrhage

	Overall (n=104)	Clazosentan group (n=43)	Conventional group (n=61)	p-value
Female sex, n (%)	78 (75)	37 (86)	41 (67)	0.029
Age, median years (range)	62	60 (21–89)	63 (24–90)	0.611
Past history				
Hypertension n (%)	39 (38)	16 (37)	23 (38)	0.959
Diabetes mellitus n (%)	5 (5)	1 (2)	4 (7)	0.401
Lipid metabolism abnormalities n (%)	11 (11)	3 (7)	8 (13)	0.519
WFNS grade				
I, n (%)	24 (23)	18 (42)	6 (10)	<0.001
II, n (%)	24 (23)	13 (30)	11 (18)	0.146
III, n (%)	4 (4)	1 (2)	3 (5)	0.641
IV, n (%)	21 (20)	8 (19)	13 (21)	0.735
V, n (%)	31 (30)	3 (7)	28 (46)	<0.001
Fisher group				
1, n (%)	0 (0)	0 (0)	0 (0)	
2, n (%)	9 (9)	6 (14)	3 (5)	0.157
3, n (%)	50 (48)	25 (58)	25 (41)	0.085
4, n (%)	45 (43)	12 (28)	33 (54)	0.008
Aneurysm				
MCA, n (%)	29 (28)	12 (28)	17 (28)	0.997
ACA, n (%)	31 (31)	16 (37)	16 (26)	0.232
ICA, n (%)	31 (30)	13 (30)	18 (30)	0.937
BA, n (%)	8 (8)	2 (5)	6 (10)	0.465
VA, n (%)	4 (4)	0 (0)	4 (7)	0.140
Clipping, n (%)	57 (55)	25 (58)	32 (52)	0.918
Coiling, n (%)	47 (45)	18 (42)	29 (48)	0.566
ICH, n (%)	33 (32)	10 (23)	23 (38)	0.119
IVH, n (%)	22 (21)	6 (14)	16 (26)	0.131
Statin, n (%)	83 (80)	38 (88)	45 (74)	0.131
Cilostazol, n (%)	98 (94)	40 (93)	58 (95)	0.689
Ventricular drainage n (%)	20 (19)	5 (12)	15 (25)	0.099
Spinal drainage n (%)	47 (45)	22 (51)	24 (41)	0.304

Efficacy endpoints

The postoperative outcomes are shown in Table 3. The incidence of DCI related to cerebral vasospasm was 2.3% in the clazosentan group and 4.9% in the conventional group, with no significant difference. The incidence of moderate or severe cerebral vasospasm was 7.0% in the clazosentan group and 9.8% in the conventional group, with no significant difference. The frequency of rescue therapy was 2.3% in the clazosentan group and 3.3% in the conventional group, with no significant difference. However, the incidence of patients with mRS ≤3 at discharge was 72% in the clazosentan group compared with 48% in the conventional group, indicating a significantly better prognosis in the clazosentan group. The median hospital stay was 35 days in the clazosentan group and 50 days in the conventional group, with no significant difference.

**Table 3:** Postoperative outcomes Data were expressed as median (interquartile range) for continuous variables and frequencies (%) for categorical variables. The Mann–Whitney U test was used to analyze continuous variables, and Fisher’s exact test and Pearson’s chi-square test were used for categorical variables. DCI: delayed cerebral ischemia, mRS: modified Rankin scale

	Clazosentan group (n=43)	Conventional group (n=61)	p-value
Efficacy endpoints			
DCI related to cerebral vasospasm, n (%)	1 (2.3)	3 (4.9)	0.641
Moderate or severe cerebral vasospasm, n (%)	3 (7.0)	6 (9.8)	0.733
Rescue therapy, n (%)	1 (2.3)	2 (3.3)	1.000
mRS ≤3 at discharge, n (%)	31 (72)	29 (48)	0.013
Hospital stay, days (range)	35 (14–91)	50 (15–149)	0.079
Safety endpoints			
Symptomatic pulmonary edema, n (%)	15 (34.9)	8 (13.1)	0.008
Mortality, n (%)	0 (0)	1 (1.6)	1.000

Safety endpoints

The frequency of symptomatic pulmonary edema was significantly higher in the clazosentan group, 34.9%, compared to 13.1% in the conventional group. No significant difference was observed in the mortality rate between the clazosentan and conventional groups (0% vs. 1.6%, respectively).

## Discussion

We investigated the effectiveness and safety of clazosentan in patients with aSAH who were treated at our institution. A search on PubMed using the keywords “clazosentan” and “fasudil hydrochloride hydrate” retrieved only three reports, those by Mochizuki et al. [9], Sakata et al. [10], and Maeda et al. [11]. Since the report by Maeda et al. was a study comparing the conventional protocol with a combined protocol of clazosentan and fasudil hydrochloride hydrate, it was excluded here. Both reports, those by Mochizuki et al. and Sakata et al., indicated that moderate or severe cerebral vasospasm incidence was significantly lower in the clazosentan group than in the conventional group. Regarding the incidence of DCI associated with cerebral vasospasm, Mochizuki et al. reported a significantly lower incidence in the clazosentan group, whereas Sakata et al. found no significant difference. Both were single-center, retrospective studies with variations in patient backgrounds, treatment strategies, and fluid management, making direct comparison difficult. In the present study, the incidence of DCI related to cerebral vasospasm was lower in the clazosentan group than in the conventional group, but the difference was insignificant. However, the incidence of moderate or severe cerebral vasospasm was comparable. Unlike the previous two studies, our study noted a high co-administration rate of cilostazol, a selective phosphodiesterase 3 inhibitor with cerebral arterial vasodilatory effects, in both the conventional and clazosentan groups, which cannot be overlooked [12].

Clazosentan can significantly increase the risk of pulmonary edema, thereby adding to the neurologic pulmonary edema caused by aSAH, which occurs in 2-31% of cases [8]. Although ETA blockade by ETA antagonists conversely activates ETB, such intervention could indirectly increase vascular permeability and induce excessive fluid retention [13]. This could explain the mechanism by which clazosentan aggravates pulmonary edema. Regarding fluid management, in the clazosentan group, fluid balance was checked every four hours from admission to maintain a total balance between −500 and +500 mL. This included appropriate fluid loading and diuretic administration. Mochizuki et al. managed fluid balance by checking every six hours, targeting +500 mL/day, whereas Sakata et al. maintained a protocol of maintaining a fluid balance within 0−500 mL. Despite stricter fluid restrictions than traditional methods, the three studies, including ours, reported a significant increase in pulmonary edema cases, indicating a need to modify the fluid balance management protocol. Akamatsu et al. reported the efficacy of combining loop diuretics with clazosentan in patients with aSAH [14]. Specifically, they compared two groups: one in which diuretic use was withheld unless oxygenation deteriorated due to fluid overload and another in which furosemide was administered when the 12-hour fluid balance exceeded 1000 mL. They found that the latter group demonstrated significantly fewer cases of symptomatic pulmonary edema [14]. Kinoshita et al. reported that combining hemodynamic monitoring systems, echocardiographic findings, and patient weight gain is effective for diagnosing clazosentan-associated pulmonary edema and achieving optimal fluid management [15]. In our study, many cases of pulmonary edema were observed early when using clazosentan. Still, only one case has been observed in 2024, indicating that symptomatic pulmonary edema may decrease with usage frequency.

This study showed improved clinical outcomes with clazosentan treatment. Although no significant reduction was observed in DCI with clazosentan use, the mRS at discharge was significantly better in the clazosentan group. However, this result should be interpreted cautiously, as the clazosentan group included mild cases (WFNS grades I and II). In contrast, the conventional group included more severe cases (WFNS grades IV and V), indicating an impact on the mRS at discharge. Further studies with larger sample sizes and the implementation of multivariate logistic analysis could verify the effects of clazosentan on the mRS at discharge. Similar to our findings, Sakata et al. found that the clazosentan group had significantly more favorable mRS outcomes at discharge [10]. However, Mochizuki et al. found no significant difference in mRS three months after discharge [9]. Because we investigated the clinical outcomes only at discharge, further studies are warranted to assess the impact of clazosentan on long-term clinical outcomes.

Limitations

This study has several limitations. First, it is a retrospective, single-center study. A large, multicenter, prospective cohort trial investigating clazosentan versus conventional management protocols would be beneficial. Second, this study only presents short-term clinical outcomes. Long-term follow-up would demonstrate the long-term clinical outcomes. Third, infusion management differed between the two protocols, which might have influenced the outcomes. Fourth, vasospasm stenosis rates were measured manually by evaluating the major cerebral arteries. The use of quantitative evaluation software could provide a more accurate assessment of the incidence of cerebral vasospasm. Fifth, between the two groups, significant differences in WFNS grades (more severe cases in the conventional group) and Fisher group 4 distribution introduce selection bias. Multivariable regression or propensity score matching is critical to adjust for confounding factors (e.g., WFNS severity).

## Conclusions

We compared the treatment outcomes between postoperative use of conventional (fasudil hydrochloride hydrate) and clazosentan treatment for aSAH. In this study, clazosentan use resulted in a lower frequency of DCI than fasudil, but the difference was insignificant. However, clazosentan can potentially cause a significant increase in the frequency of symptomatic pulmonary edema. Due to numerous limitations in this study, we cannot make definitive recommendations regarding the choice between clazosentan and fasudil hydrochloride hydrate. Further research with larger sample sizes and matched baseline characteristics is warranted to evaluate intergroup outcomes and assess long-term clinical results.
